# Neurophysiology of Milk Ejection and Prestimulation in Dairy Buffaloes

**DOI:** 10.3390/ani12192649

**Published:** 2022-10-02

**Authors:** Fabio Napolitano, Ada Braghieri, Andrea Bragaglio, Daniela Rodríguez-González, Patricia Mora-Medina, Marcelo Daniel Ghezzi, Adolfo Álvarez-Macías, Pamela Anahí Lendez, Emilio Sabia, Adriana Domínguez-Oliva, Joseline Jacome-Romero, Daniel Mota-Rojas

**Affiliations:** 1 Scuola di Scienze Agrarie, Forestali, Alimentari ed Ambientali, Università degli Studi della Basilicata, 85100 Potenza, Italy; 2 Department of Veterinary Medicine, University of Bari A. Moro, 70010 Valenzano, Italy; 3 Neurophysiology, Behavior and Animal Welfare Assessment, DPAA, Universidad Autónoma Metropolitana (UAM), Mexico City 04960, Mexico; 4 Department of Livestock Sciences, Universidad Nacional Autónoma de México (UNAM), FESC, Mexico City 04510, Mexico; 5 Animal Welfare Area, Faculty of Veterinary Sciences (FCV), Universidad Nacional del Centro de la Provincia de Buenos Aires (UNCPBA), University Campus, Tandil 7000, Argentina; 6 Faculty of Veterinary Sciences, Veterinary Research Center (CIVETAN), Universidad Nacional del Centro de la Provincia de Buenos Aires (UNCPBA), CONICET-CICPBA, University Campus, Tandil 7000, Argentina; 7 Faculty of Science and Technology, Free University of Bozen-Bolzano, Piazza Università 5, 39100 Bolzano, Italy

**Keywords:** *Bubalus bubalis*, anatomy, mammary gland, lactation curve, calving

## Abstract

**Simple Summary:**

In dairy buffaloes, the efficiency of the milking process mostly depends on the anatomical and physiological differences found in this species when compared to cattle from the genus Bos. Milk quality is influenced by individual and environmental characteristics such as health, diet, handling, and the use of exogenous hormones such as oxytocin, elements that can alter the dam’s performance. The present review aims to integrate the anatomical characteristics of the mammary gland and the neurophysiology of milk ejection to understand the milking capacity of the water buffalo.

**Abstract:**

The present review aims to integrate the anatomical characteristics of the mammary gland and the neurophysiology of milk ejection to understand the milking capacity of the water buffalo. Since one of the main uses of this species is milk production, this article will analyze the controversies on the use of oxytocin as a stimulant during milking as well as the existing alternatives that farmers apply to promote correct stimulation during milk letdown. According to the available literature, the efficiency of the milking process, the quality of the milk, and the health of the animals are elements that require the consideration of species-specific characteristics to enhance the performance of buffaloes. The incorporation of technological innovations and competitive strategies could contribute to a better understanding of water buffalo in the milk industry.

## 1. Introduction

Buffalo milk (*Bubalus bubalis*) has earned prestige in numerous areas of the world due to its properties as food for human consumption, as it is a high-quality product with desirable fat content (average 7–8%) that can be obtained with low investments in resources and in environmental conditions where other milk-producing species give only modest yields. Consequently, it has shown optimal performance in industrial processes such as transformation into derived products such as cheese, cream, and butter [1,2,3,4,5]. These results have led to the consolidation of buffalo herds worldwide, which, in 2018, reached 206 million head, 97.57% of which are on the Asian continent [6]. Regarding human consumption, buffalo milk is deemed as a valuable food; indeed, in recent years, it has risen to become the second-most consumed milk on the planet, supplying around 15% of total supplies in 2020 [7]. The knowledge and understanding of the anatomical, behavioral, adaptive, productive, and reproductive characteristics of the dairy buffalo dam is crucial for determining adequate handling methods that will ensure success in the breeding, raising, and exploitation of this species, while respecting the welfare of these animals [2,8,9]. 

This situation has led many researchers to broaden their knowledge of buffalo production focused on obtaining milk. Their studies have covered such key topics as describing the anatomy of the buffalo’s mammary gland, analyzing the neurophysiology of milk ejection, and evaluating the advantages and disadvantages of these aspects. This requires understanding and conducting follow-up on the factors that affect the milking capacity of the dairy buffalo, with the goals of achieving greater efficiency in the processes involved and reducing the milking times and costs [10,11].

One specific challenge analyzed is the greater stimulation time required prior to milk ejection when milking buffaloes, as this activity occupies around 50% of labor time on milk production units. Strategies have been explored to reduce the time spent on this activity. Milking requires supporting the calf, providing feed, and having milkers perform tactile, visual, and auditory stimulation in the form of massages. In addition, water buffaloes require relaxed surroundings, so it is important to avoid emitting loud sounds that can trigger synthesis and the secretion of adrenaline into the bloodstream, which inhibits oxytocin circulation [12]. In addition to these strategies, some producers administer exogenous oxytocin before milking, though this practice is subject to controversy due to contrasting reports on its impact on short- and long-term productive reproductive parameters [13,14,15].

The objective of this manuscript is to describe the anatomical characteristics and neurophysiological pathway that allow for milk ejection in dairy buffaloes. The factors that limit or favor correct milk ejection by fostering adequate flow during milking will be identified. In addition, scientific findings on the use of exogenous oxytocin as a stimulation method prior to milking to ensure complete emptying of the udder will be discussed in detail.

## 2. Materials and Methods

**Figure 1:**
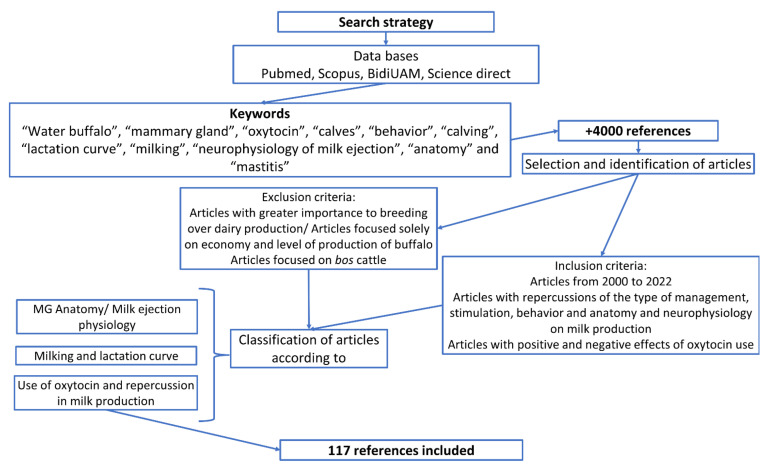
The methodology applied in this manuscript with the selection and exclusion criteria focused on fulfilling the objective of this manuscript.

This study was based on an extensive review of the literature that focused on the most recent publications that have examined the information that contributes to a better understanding of the behavior of the water buffalo during milking, in which anatomical and neurophysiological aspects must be considered, recognizing the specific needs of the species for the application of practices during manual and mechanical milking that propitiate an adequate lactation and productivity curve. The search methodology is illustrated in Figure 1.

## 3. The Mammary Gland: Pros and Contras Advantages and Disadvantages for Milk Ejection 

The mammary gland (MG) is composed primarily of epithelial cells and adipocytes of exocrine and sebaceous origin [16]. It is responsible for producing and secreting milk colostrum during lactation [17], to nourish neonates from the first hours of life with colostrum, an essential stage for the transfer of passive immunity [18], and nutrients necessary for survival including protein, fat, vitamins, and minerals [19,20]. Later, modifications of the MG are observed during the process of lactogenesis as the gland undergoes a transition from providing colostrum to milk production and the amount of immunoglobulins decrease gradually [21]. This transition reflects the changing nutritional needs of the growing calf, but the dam’s milk continues to provide the nutrients the calf requires to maintain a good state of welfare and progressive growth [22,23].

**Figure 2:**
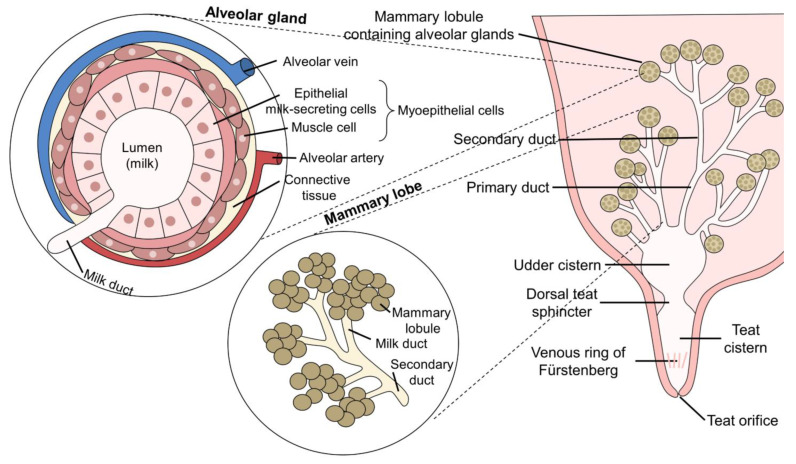
Anatomy of the water buffalo’s mammary gland. Sagittal cut of a mammary complex showing the system of lobules with their alveolar glands, enlarged alveolar glands, excretion ducts, and milk storage area.

In addition to the compositional changes in the liquids secreted, the MG undergoes various modifications during the productive life of the dairy buffalo cow from puberty through the onset of the reproductive cycle, gestation, birth, lactation, and, finally, involution during the dry period. This evolution entails a whole series of anatomical, physiological, biochemical, and neuroendocrine modifications [24]. The MG is made up of four complexes sustained by a suspensory ligament in the abdomen. The four complexes consist of a glandular body and teat [25]. Observations show that the caudal quarters have the greatest development of the mammary tissue compared to the forequarters, with an average length of 3.7 ± 0.2 and 3.0 ± 0.1 cm, respectively [26], supplying over 50% of the total milk production [27]. The MG of buffalos differ in some features from the udder of bovine dairy cattle, as it has a narrower teat canal, a thicker layer of muscle around the sphincter, more blood vessels and nerve fibers, and a smaller sphincter opening (Figure 2) [28,29].

These characteristics provide productive advantages such as a lower risk of pathologies such as mastitis [30], but some disadvantages have also been detected. The latter include the ratio of the cistern of the MG to the alveolar region (20:80), though this can largely be resolved by performing more intense stimulation accompanied by methods to optimize milking time and improve the peaks that occur along the lactation curve. These measures can improve economic returns [31], as will be elucidated throughout this article.

### 3.1. Influence of Anatomical Characteristics on Milking Capacity and the Lactation Curve

Milking capacity refers to an animal’s faculty to eject milk in response to an adequate milking technique [10]. Certain handling procedures and strategies can make milking procedures more efficient, thereby reducing the costs on milk production units, though it is necessary to constantly monitor productive characteristics and the technical parameters involved in the management of this process [32]. It has been estimated that milking takes up 50% of labor time in milk production units, so it is necessary to identify elements that improve this routine such as stimulating milk ejection, achieving an uninterrupted milk flow curve, and lessening the duration [28,32]. Goals involve avoiding sub-optimal milking and overstimulating the udder, as this can cause lesions. Milking capacity, therefore, is intimately related to the milk flow curve and hard data of the lactation curve.

The milk flow curve in the dairy buffalo cow has several phases, from the initial milk ejection through stimulation to obtaining a constant flow of milk. The plateau phase—marked by constant milk flow—is followed by a stage in which flow decreases until, finally, the blind phase occurs with the end of ejection, ideally, after a complete emptying of milk from both the cistern and alveolar gland [10]. The characteristics of the buffalo’s mammary complex include greater resistance of the muscle walls of the teat [2,33,34,35]; thus, greater vacuum pressure is required during mechanical milking to open the teat canal and reach the plateau phase. 

This background indicates the need to pay special attention to the machinery used for milking and to prior stimulation, since delays in milk ejection tend to reduce milking capacity [33], which can also be perturbed by the anatomy of individual dairy buffaloes, the stage of lactation, parity, conformation of the udder, and breed [12,33,34,36,37]. On the topic of birth number, Patel et al. [38], analyzed milking times in Mahesana buffaloes, focusing on the time that elapsed from the stimulation applied to the onset of milk ejection. They found that the time from stimulation to milk ejection decreased with a higher parity. Their study analyzed female buffaloes during their first (L1), second (L2), third (L3), and fourth lactations (L4), observing a maximum time of 71.15 s in the L1 animals versus a minimum time of 56.65 s in the L4 dams, for a gradual reduction of 3–10% between calving, differences that were statistically significant (*p* < 0.01). The impact of breed on milking capacity and the lactation curve of Italian Mediterranean buffaloes has shown percentages of cisternal fractions distinct (7.6%) [39] from those of Murrah buffaloes (4.9%) [35], which affects the re-stimulation time that dams require before the onset of milk ejection.

With respect to the total lactation curves and milking capacity, there are reports of average milking times of 11.29 min [10], though the authors emphasized that this time can increase if no previous stimulation is performed. Regarding the time of stimulation, a report on Mediterranean buffaloes mentioned 133 ± 14 s [36] and the estimated duration of 105–99.6 s, depending on the hour of milking (morning vs. afternoon) [40]. A report on the Murrah breed provided an estimated time of 69–154 s. The authors attributed the differences to the type of rations offered to each group and the hour at which milking was performed [35]. 

Commonly, reports indicate that milking time can be increased by 3 to 7 min when the stimulation is not appropriate by the handlers [36]. With respect to the lactation curve, the reported values range from 240–270 days [41,42,43,44], with a daily production of ~4–5 kg for Mediterranean buffaloes (data vary with different types of production, milking capacities, and milking methods) [2,3,45], and a total production of 1300–4500 L per lactation [46].

### 3.2. Frequency of Mastitis in Dairy Buffaloes and Its Relation to the Anatomy of the Mammary Gland

Mastitis is a disease mainly caused by bacterial and fungal infections whose manifestations include an increase in the total somatic cell count (SCC) [47]. It is one of the diseases that most frequently affects ruminants—both large and small—on milk production units [48]. Cases may be clinical or subclinical [49]. In the former, mastitis can be diagnosed by physical changes in the milk produced, which may show coagulation, and by inflammation of the quarters of the MG [50]. Subclinical cases require closer monitoring by handlers since they do not present obvious signs, but may actually be more prevalent [51]. Both types can have severe economic repercussions. For example, Guimarães et al. [52], in a study carried out on Holstein cattle raised in tropical conditions, calculated an impact of USD 19,132.35 due to mastitis over an 11-month period, based on the analyses of 142 lactating females. The authors related mastitis to the slaughter of cows (39.4%) and decreased milk production due to clinical (18.2%) and subclinical cases of the disease (32.3%). Other authors [53] investigating the environmental impact of dairy cattle (Holstein also) in the Mediterranean scenario tried to assess lost incomes due to the high slaughtering percentage of cows, mainly culled for mastitis and lameness.

Reports on dairy buffaloes from Pakistan’s livestock industry suggest that mastitis is the pathology that generates the greatest economic losses due to a prevalence of 11% in one study of 928 dams [54]. The principal microorganisms that cause intramammary mastitis infections, with repercussions that include discarding milk with high SCCs, are *Staphylococcus aureus* and *Streptococcus agalactiae* [30,55]. In their study, Lakshmi et al. [55] reported the prevalence of clinical and subclinical mastitis of 3.1 and 1.7%, respectively, attributing high SSCs to risk factors such as parity number (greater parity bringing a higher propensity). They also found that mastitis occurred most often during the first stage of lactation, the final phase of the dry period, and in the more productive animals; in addition, the type of bedding used with the buffaloes affected this pathology, as cement and dirt floors had the most pronounced impact.

Sharma and Shindhu [56] conducted a study on mastitis in Murrah buffaloes, reporting that 51.6% of the animals observed were positive, 36.3 and 63.7% for clinical and subclinical cases, respectively. The etiological agents most often identified were, once again, *S. aureus* and *S. agalactiae*, contagious microorganisms that cause acute mastitis, which damages the mammary tissue, thus impacting the welfare of the infected animals and the quality of their milk [49,57,58,59]. 

Considering the significant impact of mastitis on milk-production units worldwide, Guccione [60] found that certain morphophysiological differences between the mammary system of dairy buffaloes and cattle (*Bos taurus, Bos indicus*) resulted in lower incidences of mastitis in the former. As above-mentioned, this is due to the buffaloes’ longer teats with narrower canals, which act as barriers to block the entry and subsequent proliferation of microorganisms [61]. Despite these anatomical advantages, it is necessary to be aware of certain practices that promote the incidence of mastitis in dairy buffaloes such as inadequate handling routines by operators during manual and mechanical milking (e.g., inadequate maintenance of the teat cups, deficient hygiene and disinfection practices, and incomplete emptying of the udder) [3,13,48,49].

## 4. Neurophysiology of Milk Ejection

Once milk is produced by the secretory cells of the MG, it is stored in the alveolar gland of the udder. Estimates for dairy buffaloes indicate that 92–95% of milk remains in the alveolar compartment and only around 5% is stored in the cistern. These figures are in contrast to those for dairy cattle, where as much as 20% of the milk is stored in the cistern and ejected before the onset of the tissue contractions that trigger milk ejection by oxytocin [13,62,63].

**Figure 3:**
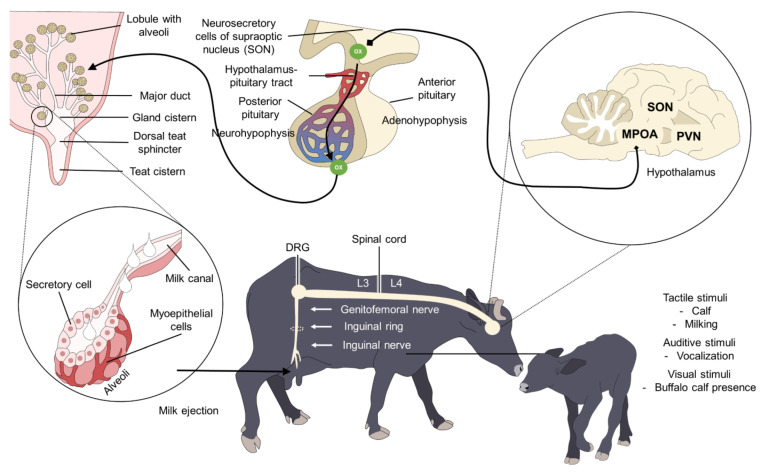
The neurophysiology of milk ejection in dairy buffalo cow. Tactile stimulation of the MG is transformed into nerve impulses that are transmitted by the inguinal nerve to the inguinal ring, which continues as the genitofemoral nerve whose roots enter lumbar pair 3° and 4° (L3, L4), reaching the ganglion of the dorsal root (DRG). There, the impulses are modulated and transferred by the ascending pathway to the cerebral nuclei of the hypothalamus (PVN, SON) and then to neurohypophysis for oxytocin (Ox) synthesis. After its release, oxytocin is stored in the neurohypophysis and later secreted into the bloodstream where it reaches the Ox receptors in the MG and contracts alveolar tissue cells to trigger milk ejection. MPOA: medial preoptic area; PVN: paraventricular nucleus; SON: supraoptic nucleus.

Milk ejection is an innate neuroendocrine reflex that responds to tactile stimulation of the teats upon the activation of receptors that are sensitive to the pressure stimulated through the inguinal canal to the lumbar nerves that connect to the dorsal roots of the spinal cord [64]. From there, a signal is transmitted to stimulate the supraoptic (SON) and paraventricular nuclei (PVN) of the hypothalamus. This, in turn, activates the neurohypophysis to release oxytocin into the bloodstream from its axonic terminals [65]. Oxytocin reaches its receptors in the MG to trigger contractions of the myoepithelial cells in the alveolar tissue and mammary ducts, thus promoting contraction of the alveolar gland. This increases intra-alveolar pressure, which brings on the shortening and thickening of the ducts in the mammary complex due to the contraction of the epithelial cells to decrease resistance to milk flow and allow alveolar milk to move into the cisternal compartment and then the teat for ejection (Figure 3) [3,15,66,67,68,69]. 

Prolactin and cortisol are secreted with oxytocin. In conjunction with the growth hormone, prolactin influences milk synthesis. The role of cortisol remains unclear. However, it has been associated with disturbed milk ejection as a result of adrenal cortex activity, low milk production, and higher SCC [70], and may influence the epithelial tight junction of the mammary gland in dairy cattle [71]. Other authors have stated that this hormone does not have an influence on the central inhibition of milk ejection [72] and that physiological increases occur during routine machine milking [73] and during suckling [74]. It is important to emphasize that the timely elimination of milk requires that concentrations reach the basal levels, a process that can be favored by a series of procedures performed before milking to induce a pre-stimulus that facilitates oxytocin release and, as a result, milk ejection. If this process is dysfunctional, one option is to apply exogenous oxytocin [75]. 

Thomas et al. [75] explored the role of oxytocin in milk ejection in six female Murrah buffaloes divided into three milking groups (two animal per group): (1) without pre-stimulation; (2) after one minute of pre-stimulation; and (3) after pre-stimulation with simultaneous alimentation. They detected that ejection occurred significantly earlier (*p* < 0.05) in treatment 3 (2.50 min) than treatments 2 (5.10 min) and 1 (6.33 min), but in all three groups, they observed an increase of >3–5 ng/L in the oxytocin concentrations during ejection. 

This result was related to the time required to reach the required plasma oxytocin concentration, which was significantly lower (*p* < 0.05) with higher oxytocin concentrations in group 3, at 96.8 ng/L, between minutes 2 and 8, compared to treatments 2 and 3, where times ranged from minutes 1 to 16, and concentrations were just 37.7 and 16.3 ng/L, respectively. These authors further reported that oxytocin concentrations increased tenfold during mechanical milking in treatment 3 compared to the basal levels and the other treatments. These data agree with other studies that determined the basal oxytocin levels in a range of 4.8–6.7 ng/L, but that the maximum concentrations could reach 90 ng/L, with ejection latencies of 2–10 min after manual stimulation. As a result, milking times are considered to be longer [36] due to the morphological characteristics of the dairy buffalo, mainly because the muscle sphincter around the teat canal is thicker than in bovines [76].

The previous description underscores the importance of the relation between oxytocin concentrations and milk ejection as well as the need to stimulate the buffalo’s teats before milking and to offer food. According to Bruckmaier and Hilger [34], oxytocin concentrations can be maintained or increased by stimulating the teat, thus impacting milk ejection, though this effect may vary in relation to the female’s productive phase.

Other authors have remarked that the ejection reflex is similar in female bovines and buffaloes due to the constant, complete emptying of the udder, which depends on the secretion of oxytocin throughout the milking process. However, since these two species have morphological differences, there may be certain particularities in the milk ejection of buffaloes that must be taken into account to ensure efficient milking [77] such as adapting the level of emptying and the frequency and proportion of pulsations during mechanical milking [33] as well as prolonged stimulation of the teat before attaching the teat cup [77]. 

### Alterations of Milk Ejection

Milk ejection can be affected at two levels. The first is the central level due to the deficient release of oxytocin from the neurohypophysis. This can be resolved by administering supraphysiological amounts of exogenous oxytocin before or during milking [78]. Boselli et al. [79] observed that dairy buffaloes with unstable oxytocin levels were normally given 20 UI, and 10 min later, milk began to flow. The second condition involves alterations detected at the peripheral level, despite normal oxytocin concentrations, due to an inadequate response of the oxytocin receptors in the MG; this state cannot be resolved by applying exogenous oxytocin [73].

The central inhibition of oxytocin secretion has been associated with several factors including the change from natural lactation to machine milking, unfamiliar surroundings, or a phase of estrus in the female that culminates in the release of ß-endorphin, which inhibits oxytocin release, and therefore milk ejection [80]. Dairy buffaloes are sensitive to changes in both milking routines and the immediate environment, so if they are stressed, frightened, or suffering, their organism will release adrenaline, which interferes with nerve impulses in the SON and PVN and, as a result, affects oxytocin release [64]. In addition, the oxytocin receptors in the alveolar myoepithelial cells can be blocked, inducing vasoconstriction of the blood vessels that impedes oxytocin from reaching the udder [36]. This condition, combined with inadequate stimulation before milking, can result in delayed milk ejection (DME) and a bimodal milk flow in which the flow is interrupted after the extraction of the cisternal milk, but before the ejection of the alveolar milk into the cistern during the inclination phase. This results in unsatisfactory milking, health problems that affect the teats, and reduced milk production [81].

Delayed milk ejection affects the health of the udder by causing a vacuum in the milk flow before the cistern and interrupting blood flow. This allows air to enter and increases the exposure to bacteria at the ends of the teats [82]. Moreover, it has been predicted that the development of DME due to inadequate milking practices can increase the concentrations of the feedback inhibitor of lactation (FIL) in the alveolar gland. This reduces milk secretion [36] due to this glycoprotein’s ability to block the biosynthesis of milk proteins. Moreover, it can induce apoptosis in the epithelial mammary cells, which reduces their sensitivity to prolactin by decreasing the number of receptors and, once again, lowering milk secretion [83]. 

## 5. Factors That Condition Affecting Milking Capacity in Dairy Buffalo

Milking capacity refers to an animal’s aptitude for performing regular, complete, and rapid milk secretion in response to adequate milking practices. Continuous monitoring is the key to achieving efficient procedures and controlling production costs. Since milking occupies over 50% of labor time on the buffalo production units, it has significant impacts on the yields and profits for producers [12]. Milk production curves are commonly used to evaluate the female buffaloes’ adaptation to the type of milking and milking routine utilized [3], both of which are intimately related to the anatomical and physiological characteristics of this species, handling routines, and udder health [12]. 

Milk production has been described as a 4-stage process. The first is the increase phase, when the alveolar myoepithelial cells are stimulated to induce milk ejection from the alveolar gland to the teat canal. The following phase, called the “plateau” phase, is characterized by a constant milk flow that reaches a peak of ejection. This is followed by the reduction phase (phase three) when milk ejection decreases. Phase three culminates in the final—or blind—phase when milk ejection ceases [3,83].

Pre-milking routines directly affect the milk ejection reflex. Observations of this period show that contact with the calf, lactation, alimentation, and manual and mechanical stimulation with rotating brushes or pulsations can serve as stimulators to improve oxytocin secretion and, hence, optimize the milking times [31,84]. Some authors have even mentioned that in dairy cows, the attachment of the teat cups without pulsations is sufficient to induce oxytocin release [85]. 

The cistern of dairy cows has a much greater volume than in dairy buffaloes, so pre-stimulation appears to be less important in that species. In buffaloes, this step is particularly important as the cavities of the cistern are empty before the onset of milk ejection. This means that the teats are also empty, so if a vacuum forms, it can lead to the collapse of the cavities. For this reason, pre-stimulation is necessary to opportunely induce milk ejection and prevent any interruption of flow in the first phase of milking [73]. 

### 5.1. Presence of the Calf during Milking in Buffalo Cows

**Figure 4:**
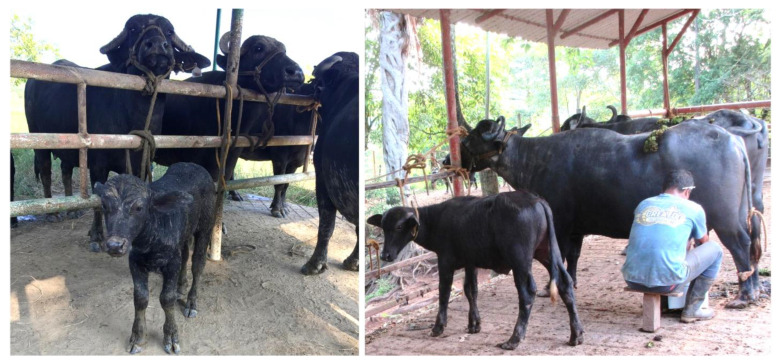
Presence of the calf during milking, a factor identified as a positive visual, tactile, and auditory stimulus for adequate milk ejection in buffalo cows (*Bubalus bubalis*).

The presence of a calf during manual milking can provide the somatosensorial stimulation required for milk ejection. The calf provides a series of visual, sensory, and olfactory stimuli that induce milk ejection (Figure 4) [3,86]. Singh et al. [87], for example, evaluated the effect of including calves in a milking study involving 24 female Murrah buffaloes. They found lower mean times to milk ejection in the group of dams with calves compared to the group without them (1.87 ± 0.13 min vs. 3.09 ± 0.26 min) as well as greater daily milk production (9.11 ± 0.16 kg vs. 6.74 ± 0.40 kg) (*p* < 0.01) and a higher rate of milk flow (1.058 ± 0.08 kg/min vs. 0.816 ± 0.09 kg/min) (*p* < 0.05). The impact of this approach also reduced the milking times in the group in which calves were used to support manual milking.

In India and Pakistan, where manual milking is the norm, producers have applied a technique that consists of allowing the calves to feed for a couple of minutes before each milking session to initiate milk ejection. On this topic, Oliveira et al. [88] state that any type of feeding—filial or otherwise—or stimulus positively impacts both the daily and total milk production, though this approach cannot be employed with dairy buffaloes if other kinds of stimulation are applied. This approach is not free of controversy since some authors argue that the presence of the calf reduces maternal prolactin excretion and, hence, milk ejection and production [36].

### 5.2. Feeding during Milking

Other studies have observed that when alterations occur during milk ejection or when lactation ends abruptly, producers may provide concentrated feed as a form of pre-stimulation (65%) instead of administering exogenous oxytocin (13%) [75]. This approach refers to the fact that manual pre-stimulation and feeding favored milk ejection in female Murrah buffaloes and culminated in a faster, more pronounced release of both oxytocin and prolactin [36], which improves milk flow [35]. Thomas et al. [89] found that female Murrah buffaloes fed 0.75 kg of concentrate accompanied by tactile pre-stimulation that consisted in washing and drying the teats showed better conditions during milking, as they presented oxytocin concentrations 3.6 times higher than the basal levels during pre-stimulation and mechanical milking, which increased as much as tenfold compared to the unstimulated dams and the condition of stimulation without alimentation. Those buffaloes also showed significantly higher rates of milk flow (*p* < 0.05) and lower cortisol concentrations (3.7 µg/L vs. 4.8 µg/L) than the dams that received only tactile pre-stimulation. This evidence suggests that using diverse stimuli may increase productivity and, at the same time, decrease the stressors that can affect milk ejection and the welfare of female buffaloes. 

### 5.3. Manual and Auditory Stimulation (Music)

Manual stimulation (MS) of the teats seems to be important for activating secretory functions in all milk-producing species [90]. Studies of cows have reported that 10–20 s of tactile stimulation is sufficient to induce oxytocin secretion and milk ejection. Female buffaloes, however, may require up to 2 min of stimulation due to the morphological differences of their udder. Similarly, there are reports that the latency from the onset of stimulation to complete ejection is 60–120 s in cows [36], but up to 3 min in dairy buffaloes [79]. On this topic, Costa et al.’s [37] study of 38 Mediterranean water buffaloes milked mechanically evaluated the effect of (i) 1–2 min of MS; (ii) exogenous oxytocin as a stimulant; and (iii) the absence of any form of stimulation before milking, in relation to morphological changes in the teat. They found that both MS and exogenous oxytocin shortened the teat canal (*p* < 0.001) compared to the non-stimulation condition, that the diameter of the cistern was greater with MS but less with oxytocin, and that the thickness of the teat wall was greater under stimulation with oxytocin but less with MS, though the latter difference was not significant. Their findings indicate that pre-stimulation induces morphological changes in the teats and in the length of the teat canal that favor milking. In this regard, Boselli et al. [79] reported a significant reduction (*p* < 0.01) of 23.1% in the length of the teat after 2 min of pre-stimulation, accompanied by changes in the thickness of the teat wall, which decreased (*p* < 0.001) by 9.28 and 18.56% after manual stimulation for 2 and 3 min, respectively, in Italian Mediterranean buffaloes.

**Figure 5:**
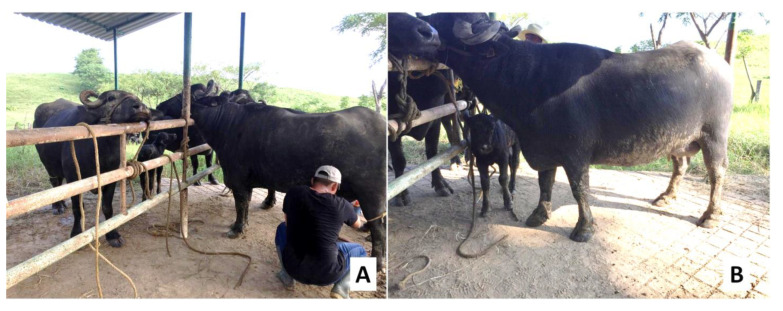
Manual and auditory stimulation during milking in dairy buffalo cows (*Bubalus bubalis*). (**A**) Manual stimulation by the milker in the form of massages to foster adequate milk ejection. (**B**) Presence of the calf to provide tactile and auditory stimulation prior to milking.

Other research has documented that MS favorably influences incidences of late ejection and latency to ejection, as observations show that after 3 min of stimulation using a routine based on cleaning the teats and udder with moist towels, milk flow began immediately upon connecting the milking machine and proceeded uninterrupted until the end of the process (Figure 5) [90]. However, the considerable amount of time that manual pre-stimulation requires has consequences for the yields achieved in the milking room because it lowers the number of dams that can be milked per hour. One option to counteract this could be pre-stimulation by means of the pulsations of the milking machine itself [91]. 

Another suggestion to mitigate the effects of neophobia associated with milking is to expose the female buffaloes to pre-recordings of the sounds typically heard in milking rooms such as those of machinery, other animals, humans, and music. This may be a good option to accustomize these animals to milking in order to attenuating problems associated with milk ejection [92]. One study of dairy cows exposed to auditory stimuli in the form of music detected voluntary approaches by the animals to the milking compartments [93]. Similarly, Ciborowska et al. [94] stated that certain music genres may function as an environmental stimulus that can relieve the negative effects of stress and pain, improve the heart rate, and reduce anxiety. Genres include classical music, lullabies, and meditation music, all of which can be beneficial for dairy cattle that may be exposed to stressors during milking. However, to date, these types of studies have been carried out only on cattle, and not on female buffaloes; thus additional research is needed on this subject [95]. 

### 5.4. Mechanical and Manual Milking

Manual milking requires applying manual pressure on the udder that imitates the kind of head-butting behavior that calves typically manifest to stimulate milk secretion [27,43]. One advantage of manual milking is that it requires minimal equipment and installations compared to mechanical milking, where the necessary installations may run from simple wooden structures to complete mechanical systems housed in concrete buildings. It is important to mention that, regardless of the milking method used, it is essential to ensure proper cleaning and disinfection of all the materials and equipment involved, accompanied by continuous training of all personnel, adequate management systems, and constant monitoring to ensure the health of the animals’ udders [33]. 

**Figure 6:**
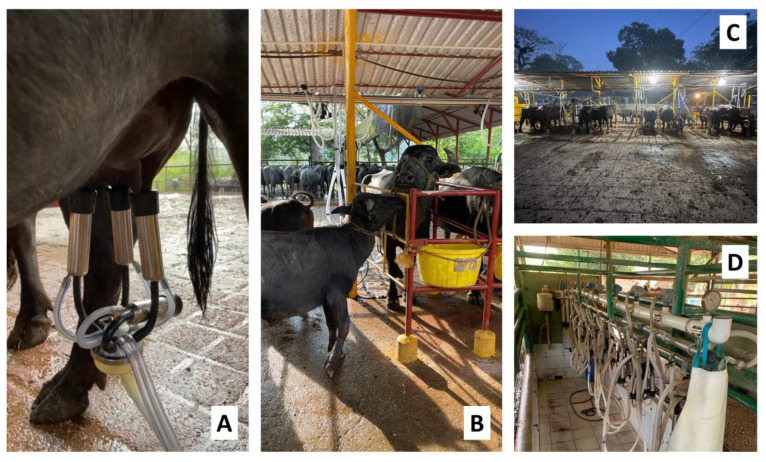
Mechanical milking in dairy buffaloes. (**A**) A mechanical milking station with a free liner for the standing calf serves as tactile stimulation for oxytocin secretion and milk ejection. (**B**) Mechanical milking station with standing calf support where tactile, auditory, and visual stimulation is allowed as well as feeders with *ad libitum* feed. (**C**) Adapted mechanical milking with space for breeding for the support and stimulation of milk ejection. (**D**) Mechanical milking parlor adapted to the characteristics of the water buffalo with the presence of feeders and without specific space for calves.

Today, mechanized milking is a leading option to increase productivity, hygiene, milk quality, and income. This method is also important because it reduces the labor costs and improves herd management [91]. These advantages have led producers to use the same technology for both dairy cattle and dairy buffalo, without necessarily taking into account the anatomical-physiological characteristics of the udders of the two species, which, as we have seen, differ significantly. The most important differences are in the volume of the tank and the size of the teats. However, the type and time of stimulation tend to be more relevant such as tactile, visual, and auditory stimuli or pelleted foods to increase productive efficiency and support rearing (Figure 6). In addition to the disparities such as those above-mentioned, the buffalo cistern is smaller than that of dairy cows, and milk from the cistern is generally not immediately available for milking as it is in cows [76].

Sannino et al. [96] conducted a study of 90 milk-producing Mediterranean buffaloes to compare an automatic milking regimen to a conventional method. They found that the former resulted in higher daily milk production (*p* < 0.001) and extended lactation times (*p* < 0.05) compared to the conventional group. They associated their findings principally with milking frequency per day and more stable milk production, respectively. Moreover, they identified significant differences in milk composition, as the product of the dams in the automatic milking group had higher concentrations of protein (*p* < 0.001) and casein (*p* < 0.01) coupled with lower bacterial counts (*p* < 0.01) compared to the milk obtained by the conventional system. 

Research shows that achieving successful mechanical milking is more complex with buffaloes than dairy cows, because buffaloes require effective stimulation before milking to avoid exposing the teats to a vacuum condition—without milk flow—for a few minutes. Reports on this topic show that, in contrast to what occurs in cows, buffaloes do not release oxytocin and, as a consequence, do not begin to eject milk when their teats are connected to the milking machine. As discussed above, female buffaloes require prior stimulation [76]. This fact was confirmed, for example, by Ambord et al. [90] in female Mediterranean buffaloes. Without prior stimulation, milk extraction was not achieved despite applying vacuum rates as high as 39 kPa. In contrast, after 3 min of pre-stimulation, the size of the teat canals decreased from 22.6 ± 2.6 to 12.9 ± 1.5 mm, a change that allowed for milk flow, even at vacuum rates below 39 kPa. Similarly, Thomas [75] affirmed that the efficiency of mechanical milking depends on opening the orifice of the teat, canal diameter, and intramammary pressure, so that when the milking machine is connected, the vacuum stretches the walls of the teat, inducing it to open. 

Singh et al. [97] worked with weaned and lactating female Murrah buffaloes subjected to manual vs. mechanical milking. They did not report significant differences with respect to the time of milk ejection between the two procedures (5.36 ± 0.21 vs. 5.82 ± 0.29 min), but cortisol and prolactin concentrations were significantly higher in the machine milked group (*p* < 0.001). The authors attributed their findings to the inadaptation of the dairy buffaloes to mechanical milking, neophobia, and difficulty in tolerating exposure to the noise produced by the milking machines.

In light of these findings, it is important that prior to milking, the handlers develop a habituation process, especially for their primiparous dairy buffaloes, since the evidence presented herein shows that these animals are highly sensitive to new environments and procedures, and that exposure to such conditions could lead them to express fear and agitated behaviors such as kicking, stomping, or frequent urination [13]. Polikarpus et al. [98] carried out a study of 16 lactating buffaloes that demonstrated that habituation for 10 days before the first day of milking including handling and cleaning of the udder significantly reduced signs of stress such as kicking (*p* < 0.01) and stomping (*p* < 0.001) during the first 20 days of milking.

During milking, producers should also consider the hierarchical, social, and preferential characteristics of different species. For example, taking care to maintain a constant order of entry into the milking room is a key point because it is part of the social behavior of various milk-producing species where social rank, state of health, and productivity are all important criteria. Other studies have reported that female buffaloes may prefer one side of the milking room over the other. Studies of dairy cows have observed that ignoring their preferences increases the frequency of stress responses. Hence, during handling, it is important to avoid interfering with the animals’ voluntary movements, as this may affect their welfare and, as a result, milk production. Additional recommendations are to reinforce milk ejection by always repeating the same milking pattern and handling of the udder, as this can improve milk quality and reduce milking times while also mitigating or eliminating stressors that can impact productivity and animal welfare [11,13]. 

One final recommendation is to ensure that milk secretion is completed when milking ends. First, this will help ensure the maintenance of milk synthesis and secretion throughout the period of lactation and, second, eliminate residual milk from the mammary gland to reduce the risks of the development of infectious processes [27].

## 6. Use and Efficacy of Oxytocin in Milk Ejection

Milk produced in the alveolar gland is stored in the alveolar cistern and MG until it is ejected by the action of oxytocin, which is synthetized in the PVN and SON, then stored in the neurohypophysis for subsequent release into the bloodstream [3,4,13,15,27,34,83,99], where it is carried to receptors in the myoepithelial cells that form the alveolar gland in each mammary complex. The objective is to trigger the contractions that eject the milk. Oxytocin (an especially important neuropeptide hormone in the milking of female water buffaloes) helps eject the milk stored in the compartments of the MG during both manual and mechanical milking. The milk in the alveolar cistern, however, can only be extracted once the myoepithelial cells begin to contract [14,63]. In scenarios where milk evacuation is incomplete during the milking procedure, both the yields and quality can be affected. It is important, therefore, to employ strategies to ensure full milk ejection from the udder. These measures may include tactile, auditory, and visual stimulation, among others [100].

**Figure 7:**
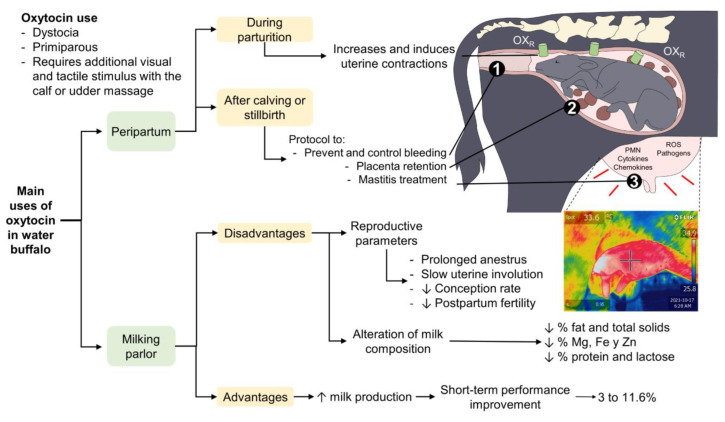
Positive and negative effects of oxytocin on buffalo milk production as reported in various studies as well as its principal uses as a tool during and after birth and in the milking process, with impacts on the characteristics of the composition of milk and its production. For example, mastitis processes can be detected with the help of non-invasive tools such as infrared thermography devices that can detect changes in the superficial temperature due to the inflammatory process of the MG. OXR: oxytocin receptors; PMN: polymorphonuclear cells; ROS: reactive oxygen species.

Consequently, applying exogenous oxytocin has emerged as a common practice among producers to obtain larger amounts of milk in less time from their herds. This measure can reduce the milking time for each animal so that more buffaloes can be milked in the same time period [3,33,89]. On this issue, Weiss et al. [101] stated that exogenous oxytocin has a positive impact on maintaining the cellular metabolism of the MG by fostering a decrease in its content. This procedure is often utilized when a buffalo’s MG becomes distended with milk since it can prevent alterations in the mammary complex and promote a healthy udder [14]. On a related topic, there are reports on the effects of exogenous oxytocin on productive and reproductive parameters when it is applied recurrently and without adequate monitoring. These effects include changes in milk synthesis and the amount of milk produced as well as differences in the nutritional characteristics and SCCs (Figure 7) [14,63,102,103,104,105]. 

### 6.1. Effect on the Synthesis and Production of Milk

During milking, dairy buffaloes show a greater sensitivity to stressful stimuli than bovines of the genus Bos. This can lead to the synthesis and secretion of adrenaline, which counteracts the production and release of oxytocin. Adrenaline also prevents adequate contractions of the epithelial tissue in the alveolar gland and can interrupt milk flow [89]. Observations of this phenomenon show that an efficient emptying of the udder using exogenous oxytocin increases the total milk produced, but other factors must be considered before deciding to intensify the frequency of milking, since the possible increase in total kilos produced may be accompanied by modifications in the composition of the milk [83].

Faraz et al. [14] compared the productivity of female Nili-Ravi buffaloes with the nutritional content of their milk due to the intramuscular administration of 20 UI of oxytocin. They reported significantly higher milk production (*p* < 0.05), ascribed to the ejection of residual milk. This measure also reduced diseases of the MG due to the complete emptying of the udder. Lollivier et al. [83] found a positive correlation between the use of exogenous oxytocin and an increase of 8% in milk production. In the study by Akhtar et al. [66], female Nili-Ravi buffaloes were given 30 UI of oxytocin via intramuscular one day before commencing milking and then during and after milk extraction. They determined that the animals that received this treatment showed differences (*p* < 0.05) in milk production with yields of 8.57 ± 0.07 vs. 8.40 ± 0.04 L for a control group. In their work, Rushen et al. [106] recorded higher milk production in Holstein cows after the administration of oxytocin compared to a control group, with values of 8.7 ± 0.8 vs. 3.2 ± 0.8 kg. Finally, Sitkowska [107] mentioned that the milk yields of female buffaloes increased with the administration of oxytocin, but that their milk presented affectations in terms of the fat and protein content.

### 6.2. Effect on Nutritional Characteristics of the Milk and Somatic Cells 

Some authors sustain that administering oxytocin does not affect milk quality [102,108]. However, other researchers evaluating qualitative parameters have found modifications due to the effect of applying exogenous oxytocin to stimulate the emptying of the udder. Faraz et al. [14] assessed the nutritional characteristics of the milk of Nili-Ravi buffaloes, and observed that the fat content decreased as the production increased, with changes from 9.47% ± 0.46 in the control group to 8.01% ± 0.04 in the group treated with oxytocin at the onset of lactation, from 9.65% ± 0.05 to 8.71% ± 0.2 in the peak period, and from 9.22% ± 0.28 to 8.81% ± 0.19 at the end of lactation. They also reported lower percentages of lactose, protein, total solids, and non-fat solids in each stage observed, with negative consequences on milk processing. Kiran [109] also observed a significant reduction (*p* < 0.05) in the percentages of fat and both non-fat and total solids in their study. They further reported significant decreases (*p* < 0.01) in Mg, Fe, and Zn content, no effects on Ca, and increased Cu and Mn content after oxytocin administration. 

Faraz et al. [14] experimented with constant oxytocin application to detect its impact on the mineral composition of milk. Their results led them to suggest eliminating this practice during milking because of its potential impact on consumers, though the same report stated that injecting oxytocin in lactating female buffaloes was not reflected in the milk content because it is a neuropeptide that is quickly degraded in intestinal digestion and not absorbed. Thus, it has no harmful effects for people who consume buffalo milk products.

The mass of milk somatic cells is almost entirely represented by white globules and epithelial cells that detach from the lining of the MG, especially in the late stages of lactation and white globules that increase in cases of mastitis infection. The SCC, in fact, is a parameter commonly used to determine the health of the MG and milk quality [110], as it provides values that are utilized in both the public and private sectors as points that companies should consider when purchasing milk and determining payment for producers in relation to the characteristics of the milk at the moment of the transaction. Variations have been observed in SCC due to the effects of oxytocin. Akhtar et al. [66] observed a significant increase (*p* < 0.05) in a group treated with 30 UI of oxytocin, with variations of 72.96 to 97.01 × 103 in the treatment group compared with the variations of 71.86 to 77.14 × 103 in the control group. Bidarimath and Aggarwal [63] observed higher SCCs in the milk of Murrah buffaloes treated with oxytocin on days 0, 15, 30, and 45 postpartum, with increases of 5.36–6.22% compared to the control group. 

### 6.3. Other Effects

In addition to the effects on the productive parameters, modifications of some indicators of the health and reproductive function of dairy buffaloes have been detected as a consequence of the constant use of exogenous oxytocin due to its action on long-term neurophysiological processes [111]. These modifications include a higher number of abortions and cases of placental retention [101,112]. Mustafa et al. [111], in Pakistan, diagnosed negative consequences of the constant use of exogenous oxytocin including a higher percentage of stillbirths (13.3%), frequencies of distocic births (71.66%), and cases of placental retention (38.33%) as well as cases of reproductive pathologies such as the formation of luteal (23.33%) and follicular cysts (26.66%), a higher incidence of repeat breeder dairy buffalo (25%), and prolonged anestrus (18.33%). Qureshi and Ahmad [112], administering 7.50 UI of oxytocin on female Nili-Ravi buffaloes, found that this treatment increased both the time for expulsion of the placenta and the postpartum ovulation interval.

Other studies have reported the negative impacts of exogenous oxytocin on the secretion of endogenous oxytocin and its action on the MG, with a decrease in cellular contractibility in the alveolar gland, which lowers milk ejection when it is withdrawn. This practice was catalogued as “harmful” when performed on a daily basis [14,15,57,83,113,114,115,116,117]. However, few articles have provided strong data on negative relations between the use of oxytocin to stimulate milk ejection and reproductive performance in the water buffalo. Therefore, additional research is necessary to obtain a greater understanding of the impact of this practice on the health of milk-producing buffaloes and the economics of production units.

## 7. Conclusions

It is imperative to obtain detailed knowledge of the key anatomical characteristics of the buffalo cows (*Bubalus bubalis*) in order to design and implement strategies to improve their productive parameters. In addition, researchers and producers have an obligation to recognize the normal neurophysiology and behavior of this species during breeding and lactation to ensure adequate herd management. More research is required to determine the impact of the constant and long-term use of oxytocin by the parental pathway on productive and reproductive parameters, understanding that it may be advisable to eliminate this controversial practice from buffalo production units and replace it with good practices during milking that favor milking capacity and the productive characteristics of the dairy buffalo with no short- or long-term affectations.

Other relevant and possible fields of study concern the relationship between improved conditions of animal welfare such as a good quality of the human–animal relationship and productivity in quantitative–qualitative terms; this could reduce the use of the practice of exogenous administration of oxytocin to buffaloes.

## Data Availability

Not applicable.
